# Continuum percolation in colloidal dispersions of hard nanorods in external axial and planar fields

**DOI:** 10.1039/d1sm01408k

**Published:** 2021-11-08

**Authors:** Ilian Pihlajamaa, René de Bruijn, Paul van der Schoot

**Affiliations:** Group of Soft Matter and Biological Physics, Eindhoven University of Technology Eindhoven The Netherlands i.l.pihlajamaa@tue.nl

## Abstract

We present a theoretical study on continuum percolation of rod-like colloidal particles in the presence of axial and planar quadrupole fields. Our work is based on a self-consistent numerical treatment of the connectedness Ornstein–Zernike equation, in conjunction with the Onsager equation that describes the orientational distribution function of particles interacting *via* a hard-core repulsive potential. Our results show that axial and planar quadrupole fields both in principle increase the percolation threshold. By how much depends on a combination of the field strength, the concentration, the aspect ratio of the particles, and percolation criterion. We find that the percolated state can form and break down multiple times with increasing concentration, *i.e.*, it exhibits re-entrance behaviour. Finally, we show that planar fields may induce a high degree of triaxiality in the shape of particle clusters that in actual materials may give rise to highly anisotropic conductivity properties.

## Abstract 2

Long rod-like particles in external planar and axial quadrupole fields display highly complex percolation behaviour, featuring multiple re-entrant effects and triaxial connected clusters.

## Graphical abstract

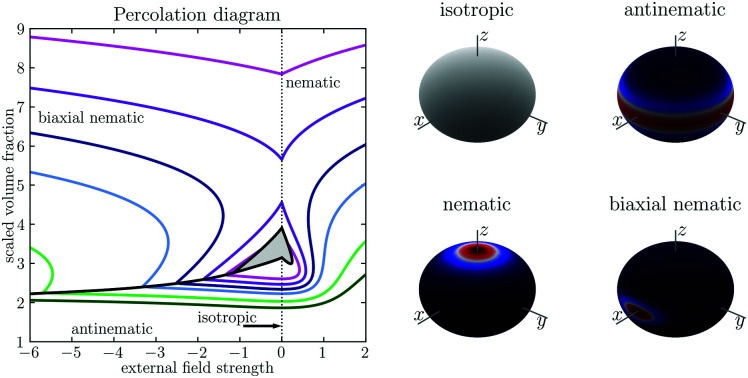

## 1 Introduction

The addition of nanoparticles such as carbon nanotubes, carbon black, silver nanowires or graphene to common plastics potentially enhances their electrical and heat conductivity tremendously.^1–9^ Indeed, the incorporation of even a relatively small amount of a carbon nanotube (or graphene) filler into an insulating polymeric host material increases its electrical conductivity by over ten orders of magnitude.^7,10^ Not surprisingly, these polymer composites have stirred up a great interest for industrial applications in photovoltaics,^11,12^ optoelectronics,^13^ gas sensing,^14^ and liquid crystalline display devices.^15^

Typically, the large effect that nanoparticles have on the conductive properties of their insulating host material is seen in the context of continuum percolation theory.^16–18^ If added in sufficient quantities, the nanofillers form a network of particles that spans the entire material. It is through this network that electricity (or heat) can be conducted efficiently. The critical packing fraction associated with the formation of this network is called the percolation threshold. Percolation theory aims to find this critical particle concentration. We note that nanofillers need not be connected physically, but transport may be mediated *via*, *e.g.*, electron tunnelling.^19^ This is commonly modelled by defining particle–particle connections *via* a distance criterion.^20^ Continuum percolation theory, typically, does not predict the actual conductivity of the resulting nanocomposite, as it is solely a geometric framework for establishing whether a macroscopic, material-spanning network exists. See, however, the work of Grimaldi and collaborators for a notable exception.^21^

Common engineering applications require that the nanoparticle loading in composites is as low as possible in order to retain the advantageous properties of polymeric materials such as their optical transparency, mechanical flexibility, and low manufacturing cost.^22^ In practice, this requires that the percolation threshold should be as low as possible. It turns out that this percolation threshold depends strongly on the degree of anisometry of the dimensions of the nanoparticles,^23–25^ scaling with the inverse of the aspect ratio.^25,26^ While this suggests that the percolation threshold can be made arbitrarily low by increasing the aspect ratio of the nanofillers, practical limits exist, as, for instance, long carbon nanotubes tend to break during processing.^27,28^

A major additional challenge has surfaced in recent decades. It turns out that the percolation threshold is not only very sensitive to the particle shape, but also to many other factors, some of which are very difficult to control consistently in experiments.^29^ Examples are the particle size polydispersity,^30–32^ inter-particle interactions^33^, spatial inhomogeneity of the dispersion^34^ as well as any degree of particle alignment caused^35^, *e.g.*, by the processing of the composite or the spontaneous formation of liquid crystalline states.^36^ These factors come into play as almost unavoidable “imperfections”, and depend non-trivially on the relevant production and manufacturing processes.^37^ It is not surprising, then, that a large body of simulation and theoretical literature has emerged, attempting to deal with such aspects of non-ideality.^36,38–46^

Specifically, Finner *et al.* have recently shown that dispersions of hard, rod-like particles exhibit a highly complex percolation behaviour when subjected to a quadrupole alignment field.^45^ In the dilute limit, the particles cannot create material-spanning clusters and less so in a alignment field. As one adds more particles, however, the average network grows, until its size diverges at the percolation threshold. At even higher densities, the work of Finner and co-workers shows the alignment induced by the external field and excluded-volume interactions can suppress percolation. To restore the percolating state, one could choose to add even more particles, regaining system-spanning networks. However, testament to the non-linearity of the interplay between the induced alignment by these two sources, predictions show that percolation can break down and form again at even higher densities.^45^

In this paper, we extend the earlier work of Finner and co-workers by investigating the impact of a so-called disorienting field.^45^ A disorienting field is a planar orienting field that aligns the particles towards a plane perpendicular to the field direction. It can for instance be the result of the extensional flow field found in a four-roll-mill setup.^47,48^ While uniaxial in orienting fields, the orientational distribution function of rod-like particles can become biaxial in planar (disorienting) fields, and, as we shall see, this turns out to have a significant impact on the percolation threshold. The difference between an orienting and a planar (disorienting) field makes itself also felt in the phase behaviour of hard rods, because the isotropic-nematic binodal ends in a critical point for the former yet in a tricritial point for the latter. We show that the strongest impact a quadrupolar field has is near the second order transition line that ends in the tricritical point. We also find that whilst corrections for finite particle aspect ratios do introduce a high density percolation threshold and quantitatively affect our predictions for the percolation threshold, the topology of the percolation diagram for low to intermediate concentrations does not change qualitatively.

The remainder of this paper is subdivided into four parts. Firstly, for completeness we devote a short section on Onsager theory in an external quadrupole field. Secondly, we apply continuum percolation theory to find under what conditions dispersions of those particles percolate. Thirdly, we investigate the effect of the aspect ratio by combining the theory presented in the previous sections with a Scaled Particle Theory. In the last section, we show that non-percolating clusters can become significantly triaxial due to the external fields by analysing correlation lengths.

## 2 Onsager theory revisited

Hard-core interactions cause rod-like particles to spontaneously align along a preferred direction called the director, if the particle concentration is sufficiently high. This was first recognised by Lars Onsager, who proposed a second virial theory to describe the transition from the isotropic to the uniaxial nematic state.^49^ In this section, we briefly review Onsager theory, applied to a dispersion of cylindrical particles subject to an external field, and summarise our numerical implementation of it.^45,47,50,51^ We refer to the works of Onsager,^49^ Frenkel,^52^ Stephen and Straley,^53^ Odijk^54^ and Vroege and Lekkerkerker^55^ for more complete treatments of the theory.

A central ingredient in Onsager theory is the orientational distribution function *ψ*(**u**) of uniaxial particles, where the orientation vector **u** can be expressed in spherical coordinates as **u** = (sin *θ* cos *ϕ*, sin *θ* sin *ϕ*, cos *θ*)^T^ with *θ* and *ϕ* the polar and azimuthal angle. We model the particles as impenetrable (sphero)cylinders with length *L* and diameter *D*. It makes sense to introduce a dimensionless concentration *c* = π*ρL*^2^*D*/4, which is equal to the product of the aspect ratio and the hard-core volume fraction in the slender-particle limit *L* ≫ *D*, which is where the second-virial approximation is believed to become exact. Here, the number density is given by *ρ* = *N*/*V*. In the current and next two sections, we assume that the so-called Onsager limit holds, *i.e.*, the particles are infinitely slender *D*/*L* → 0, and consequently, we can neglect all effects of the hemispherical end-caps. In Section 4, we lift this assumption and consider also the effects of the finite slenderness.

The particles are subject to an external quadrupole field, which, for simplicity, we assume to be oriented along the *z*-axis. It assigns an energy to each particle of the form *U*/*k*_B_*T* = −*K* cos^2^ *θ*, where *k*_B_*T* is the thermal energy and *K* is a dimensionless field strength. If *K* is positive, it is energetically favourable for the particles to align along the *z*-axis, and we call the field orientational or axial. If *K* is negative, however, the particles prefer to be aligned in the *xy*-plane, and we refer to it as disorientational or planar.

Within Onsager theory, the dimensionless Helmholtz free energy per particle reads, up to an arbitrary constant,1
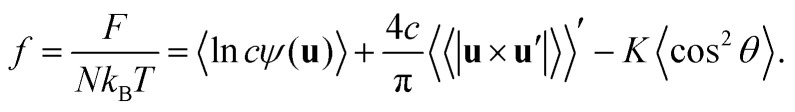
Here, we introduce a shorthand notation for the angular averaging operators 
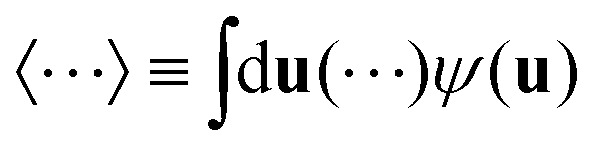
 and 
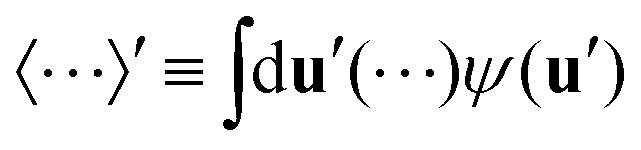
, where the integrals run over all orientations on the unit sphere. The first term of the free energy describes the translational and orientational free energy of ideal uniaxial particles, whereas second term describes the excluded volume interactions, in which2
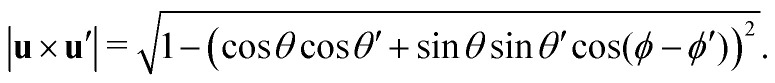
An expression for the most probable orientational distribution function can now be obtained by functionally minimising the free energy with respect to *ψ*(**u**) while enforcing its normalisation. This yields the Onsager equation3
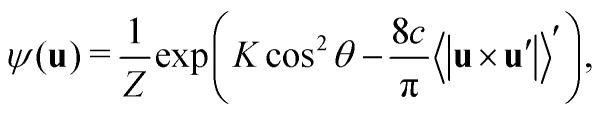
where *Z* ensures the normalisation condition is met. Eqn (3) is a nonlinear self-consistent integral equation for *ψ*(**u**) of which no exact non-trivial solution is known.

We solve eqn (3) numerically by recursive iteration, monitoring convergence by evaluating if the maximal element-wise difference between two subsequent iterations of the discretised orientational distribution function is smaller than 10^−8^. This procedure is known to be highly convergent when applied to the Onsager equation, and typically terminates within 10–30 iterations.^56^ We approximate the spherical integrals using a 131st order Lebedev quadrature, which ensures that all polynomials up to 131st order are integrated exactly.^57,58^ We find that this yields higher accuracy with fewer grid points than trapezoidal rules.^45,51,59^

**Fig. 1:**
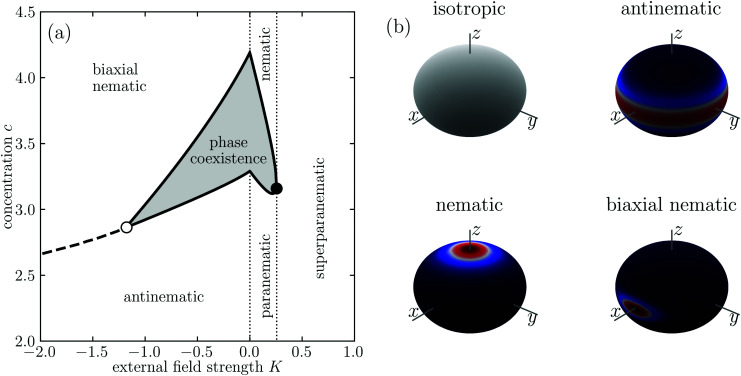
(a) Phase diagram of infinitely slender rod-like particles in an external quadrupolar field as a function of the dimensionless concentration *c* and external field strength *K*. Indicated are the different states, separated by dotted vertical lines. The isotropic state, which occurs at on the vertical line *K* = 0, *c* < 3.290, is not indicated. For sufficiently weak external fields, the dispersion separates in a high-density and a low-density phase. The region in which this occurs is enclosed by the binodals, which are indicated by solid lines. We find a critical point (solid circle) at *K* = 0.255 and a tricritical point (open circle) at *K* = −1.2. At the tricritical point, the binodals converge, and form a continuous phase transition (dashed line) between the antinematic and biaxial nematic phase. (b) The orientational distribution function *ψ*(**u**) in the isotropic, antinematic, nematic, and biaxial nematic phases. The external field axis was chosen along the *z*-axis. The paranematic and superparanematic states have similar symmetries as the nematic and are therefore not drawn independently. The colours indicate the value of *ψ*(**u**), red and blue respectively denoting a high and low probability of finding a rod with the corresponding orientation.

For sufficiently weak external fields, a coexistence region exists between a low-density disordered phase and a high-density ordered phase. For these phases to be in thermal equilibrium, the pressures *p* = −(∂*F*/∂*V*)_*N*,*T*_ and chemical potentials *μ* = (∂*F*/∂*N*)_*V*,*T*_ must be equal in both phases.^46,60^ For a fixed field strength, we can solve the resulting coupled set of equations, of which the results are shown as a function of *K* in Fig. 1. Our numerical results agree quantitatively with earlier obtained numerical values.^50,51,59^

We can distinguish between six distinct regions in Fig. 1. If the external field *K* is equal to zero, and the particle dispersion is sufficiently dilute (*c* < 3.290), then the orientational distribution function takes a constant value *ψ*(**u**) = 1/4π and the canonical (scalar) nematic order parameter *Q* ≡ 3〈cos^2^ *θ*〉/2 − 1/2 takes on the value *Q* = 0. This is the isotropic phase, not indicated in the figure. If the mean concentration falls in the range *c* ∈ (3.290, 4.191), the particles spontaneously separate into an isotropic domain of density *c* = 3.290 and a high-density nematic domain where *c* = 4.191 and *Q* = 0.7922. For *c* > 4.191, only the nematic phase survives. The location of the binodals we find is consistent with their literature values to at least three decimal places.^55^

If the external field strength is sufficiently weak, the phase transition remains first order albeit that the coexistence region shrinks as the field becomes stronger. In the case of a orienting field (*K* > 0), the binodals end in a critical point at *K* = 0.255, whereas for a planar field, they end in a tricritical point at *K* = −1.2, *c* = 2.9, *Q* = −0.25, *η* = 0.05. For *K* < −1.2, we have a second order transition between an antinematic phase and a biaxial nematic that we pinpoint by evaluating the location of the discontinuity in the derivative of the order parameter *η* ≡ 〈sin *θ* cos 2*ϕ*〉.^47^ For the antinematic phase, we have *Q* < 0 and *η* = 0, while in the biaxial nematic phase we have *Q* < 0††The order parameter, here, is defined with respect to the field direction, which for the biaxial phase is perpendicular to the director. and *η* > 0.

For weak orienting fields, 0 < *K* < 0.255, we have coexistence between a dilute paranematic phase (*Q* > 0, *η* = 0) and a concentrated nematic phase (*Q* > 0, *η* = 0). If the field is sufficiently strong, however, the first order phase transition between the paranematic and the nematic phase vanishes. The region in which this distinction is lost, may perhaps be called superparanematic (*Q* > 0, *η* = 0). The corresponding critical point is located at *K* = 0.255, *c* = 3.2, and *Q* = 0.44.^50^

## 3 Geometric percolation

To describe the percolation behaviour of a dispersion, we first need a criterion that establishes whether or not two particles are connected. To this end, we model each particle as being surrounded by a so-called connectivity shell with thickness *λ*/2. If the connectivity shells of two particles overlap, we consider them connected. This corresponds to the criterion that the surface-to-surface distance between particles is smaller than *λ*. For homogeneously dispersed rod-like particles, we can now express the weight averaged connectedness structure factor *S*^+^(**q**), in which **q** is a wave vector, as^61,62^4
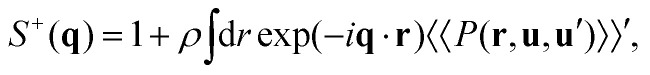
where *P*(**r**, **u**, **u**′) is the pair connectedness function, which quantifies the likelihood of finding two connected particles with a relative position vector **r** and orientations **u** and **u**′. We identify the percolation threshold with the density at which the cluster size *S* = *S*^+^(|**q**| → 0) diverges.

The common way to make headway here, is to apply the theory introduced by Coniglio *et al.*,^61^ who derived an Ornstein–Zernike equation for the pair connectedness function and the direct connectedness function *C*^+^, which in Fourier space reads5
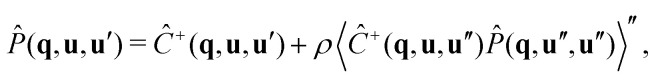
where the hats indicate Fourier-transformed quantities. Loosely speaking, *C*^+^(**r**, **u**, **u**′) can be interpreted as a measure for the likelihood of the two particles being directly connected.^63^ Hence, to lowest order in the density we may presume it to be equal to a Boltzmann factor, in which case *Ĉ*^+^ becomes equal to^39^6
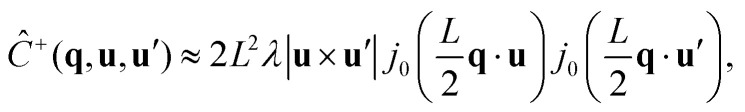
which is valid provided *L*/*D → ∞* and |**q**|*D* → 0, *i.e.*, in the Onsager limit, and formally equivalent to the second virial approximation. In eqn (6), we have introduced the zeroth order spherical Bessel function *j*_0_(*x*) = sin *x*/*x*. In the limit |**q**|*L* → 0, *Ĉ*^+^ becomes equal to the connectivity volume, that is, the volume in which a particle with orientation **u**′ can be found, such that its connectivity shell overlaps with a particle with orientation **u**.

To calculate the cluster size *S* from eqn (4), we solve the Onsager eqn (3) and the connectedness Ornstein–Zernike eqn (5) self-consistently. This we do numerically. First, we find the orientational distribution function using the method described in Section 2. The second step is to solve the integral eqn (5). To do this, we reduce the dimensionality of the problem by pre-averaging this equation over **u**′, and solving for *ĥ*(**u**) = 〈*P̂*(**0, u**, **u**′)〉′. In terms of this intermediate function *ĥ*, the connectedness Ornstein–Zernike equation reads

7 *ĥ*(**u**) = 〈*Ĉ*^+^(**0**, **u**, **u**′)〉′ + *ρ*〈*C*^+^(**0**, **u**, **u**′′)*ĥ*(**u**′′)〉,

with *S* = 1 + *ρ*〈*ĥ*(**u**)〉.

In contrast to the nonlinear Onsager equation for the orientational distribution function, eqn (7) is linear. As such, we do not need to perform a recursive iteration, but can write it as a matrix equation **h** = **b** + **Ah** and solve it straightforwardly using standard numerical methods.^64^ To turn eqn (5) into a vector equation, we introduce the discretised orientation vector **u**_*i*_ in accordance with the Lebedev quadrature that we employ for solving the Onsager equation.^58^ This allows us to introduce the elements *h*_*i*_ = *h*(**u**_*i*_), 
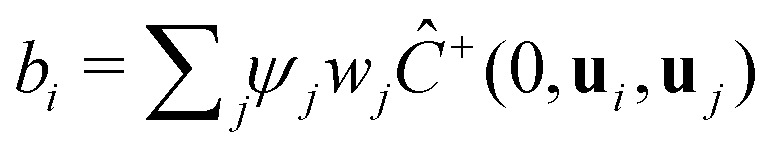
, and *A*_*ij*_ = *ρC*^+^(**0**, **u**_*i*_, **u**_*j*_)*w*_*j*_*ψ*_*j*_, defining *ψ*_*j*_ = *ψ*(**u**_*j*_) and *w*_*j*_ as the weight associated with the grid-point **u**_*j*_. The cluster size is now straightforwardly calculated from 
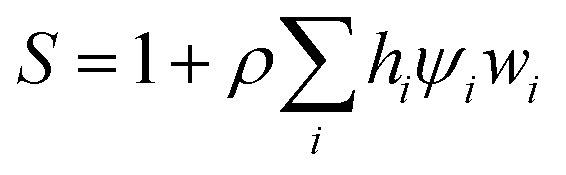
.

We locate the percolation threshold by performing a sweep of concentration *c* and field strength *K* requiring that 1/*S* = 0, which translates to the physical requirement that the average cluster size diverges at the onset of percolation.

**Fig. 2:**
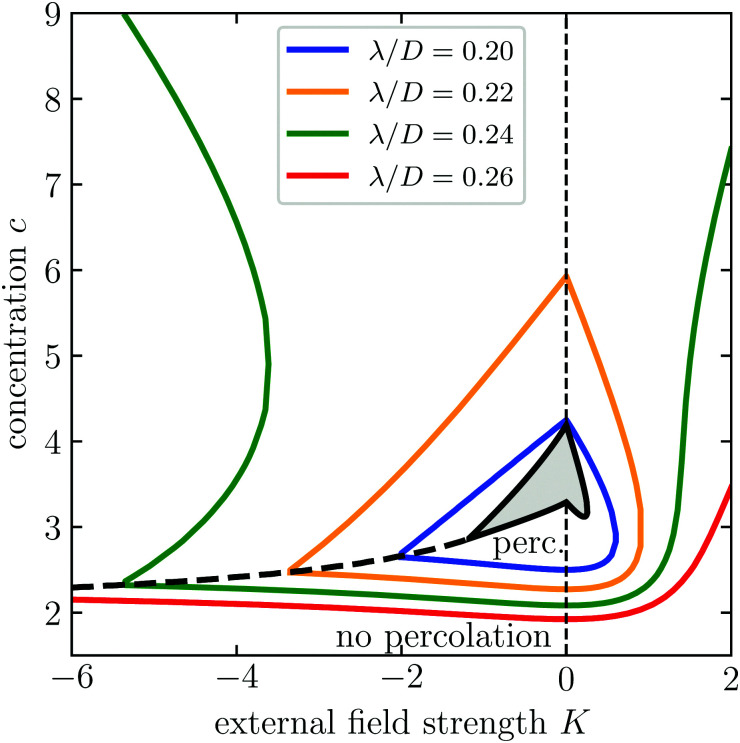
The occurrence of percolation for varying concentration *c*, external field strength *K* and connectedness shell thickness *λ*/*D*. For very low concentration, no percolating cluster exists. As the concentration increases, a percolating cluster is formed and possibly destroyed (and sometimes reformed) dependent on the value of the external field strength and the connectivity range *λ*/*D*. Also indicated in the figure are the binodals and second order phase transition computed from Onsager theory, see Fig. 1.

We perform this procedure throughout the parameter space spanned by our three parameters *c*, *K*, *λ*/*D* to obtain a comprehensive diagram that shows under what conditions percolation occurs. This diagram is shown in Fig. 2, and extends that of the earlier work of Finner and collaborators to negative values of *K*.^45^ The presence of the second order transition line, separating the antinematic and biaxial nematic phase, drastically changes the course of the percolation line in the state diagram. Indeed, the percolation line exhibits a cusp at the second order transition line. We do not find this kind of behaviour for *K* > 0, *i.e.*, for orienting fields.

From Fig. 2, we can identify four regimes depending on the value of thickness of the connectivity shell *λ*/*D*.

(1) If the connectedness shell thickness obeys *λ* < 0.15*D*, then the alignment induced by the external field and hard-core interactions prevent percolating clusters from appearing altogether. This has been shown analytically and was confirmed by Monte Carlo simulations by Finner *et al.*, in the absence of an external field.^65,66^ Noting that quadrupole fields always inhibit the formation of percolating clusters to some extent, we conclude that this finding remains valid in the presence of an external field.

(2) For 0.15*D* < *λ* < 0.2*D*, the percolation line is a closed loop, but intersects the phase coexistence region. This means that for certain values of *K* and the mean concentration *c*, the rods in the isotropic domain may percolate while in the nematic domain they do not. For sufficiently weak external fields, percolating particle clusters are formed in the dilute phases (isotropic, paranematic, and antinematic). Upon increasing the concentration or field strength, the alignment induced by either the external field or the hard-core interaction always cause the percolating clusters to eventually disconnect. This implies that we witness re-entrant percolation as a function of both the field strength, running from large negative to large positive values, and of the concentration, running from the very dilute to the highly concentrated.

(3) If 0.2*D* < *λ* < 0.236*D*, percolation islands remain to be present, but they no longer intersect the phase coexistence region, meaning that if phase separation occurs, rods in both phases percolate. Not surprisingly, re-entrance percolation survives.^45,65^

(4) For *λ* > 0.236*D*, the percolation islands fan out, and transform from islands to peninsulas, and from peninsulas to normal coastlines. Depending on the precise value of λ/*D* there may be repeated re-entrance effects, where percolating clusters form and break down multiple times. For *K* < 0, the region in which double re-entrant effects occur is notably larger than that for positive values of *K*.

Thus far, we considered the Onsager limit, that is, the limit in which the particle aspect ratio goes to infinity. Actually, the theory discussed above should be expected to become quantitatively correct for aspect ratios exceeding a few hundred.^66^ Even though that this covers many practical applications, involving, *e.g.*, carbon nanotubes, we need to ask ourselves the question if our main conclusions remain valid if we correct for finite aspect ratios. This we investigate next.

## 4 Finite aspect ratios

In the preceding sections, as already alluded to, we considered the phase behaviour and percolation of infinitely slender nanorods in an external field. The question arises in what way our predictions change if we let the particle aspect ratio decrease to more realistic values down to, say, a hundred or even twenty, more typical of colloidal systems. For this purpose, we need to include corrections to Onsager Theory of higher order in the density. We choose to make use of a renormalised second-virial approach based on Scaled Particle Theory (SPT).^45,67,68^ This approach is similar in spirit to the Parsons-Lee approach, yet has been shown to yield results that are in better agreement with Monte Carlo simulations of percolation, and in fact remains nearly quantitative, even down to aspect ratios of *L*/*D* = 5.^66,69,70^ We briefly present the main ingredients of the theory, and proceed to apply it to percolation of rods in external fields.

We consider a dispersion of spherocylinders rather than cylinders for reasons of tractability.^49^ Therefore, we take hemispherical end-caps into account, implying that the aspect ratio of the particles becomes *L*/*D* + 1. Here, *L* denotes the length of the cylindrical part of the particle and *D* its width. The volume fraction of a dispersion of such spherocylinders is given by8
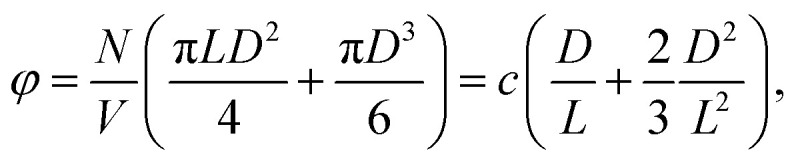
where the dimensionless concentration remains to be defined as *c* = π*ρL*^2^*D*/4.

Cotter and Wacker derived an expression for the SPT free energy of spherocylindrical particles, which is exact up to second order and approximate for all higher orders in the density.^67,71^ For a review of the theory, we refer to Lekkerkerker and Tuinier.^68^ Tuinier *et al.*^72^ show that SPT simply renormalises the concentration *c* in eqn (3) and yields9
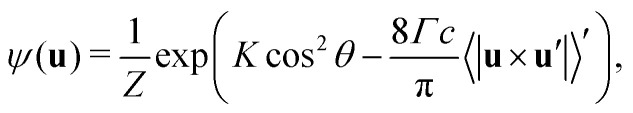
in which *Z* is again a normalisation constant, and10
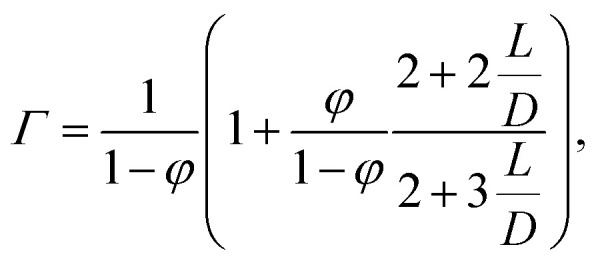
is the SPT correction factor, which depends only on the volume fraction and the aspect ratio of the particles. In the infinite aspect ratio limit, eqn (9) reduces to eqn (3) for *Γ* → 1 as *L*/*D → ∞* at constant *c*.

**Fig. 3:**
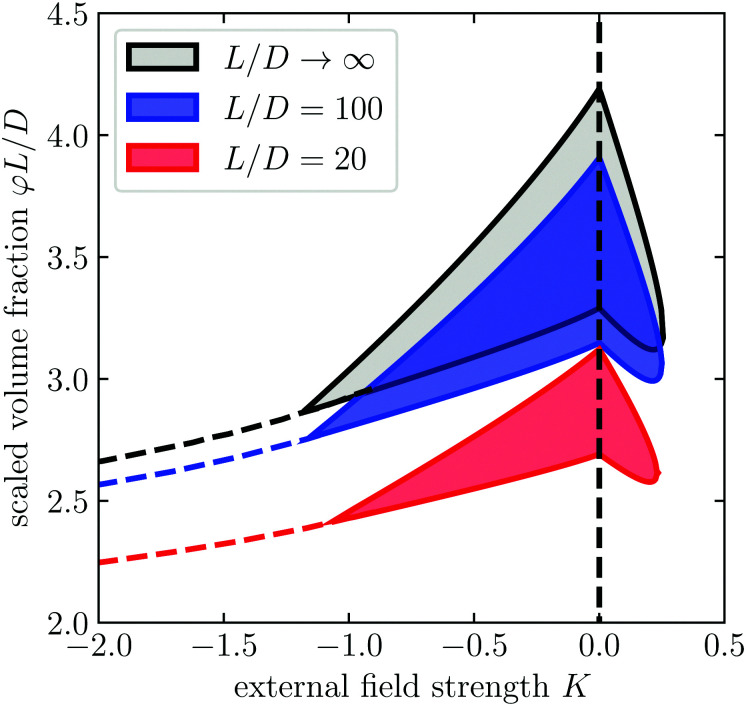
Phase diagram of hard spherocylinders with aspect ratios *L*/*D* ∈ {∞, 100, 20} in terms of a scaled volume fraction as function of the external field strength *K*. Within the enclosed region, high and low density phases coexist. We computed the location of the binodals using the Onsager free energy, with scaled particle theory corrections for finite particle aspect ratios. The terminology of the phases is unchanged and shown in Fig. 1.

Following the same procedure that we used to obtain the phase diagram for infinitely slender particles, we obtain phase diagrams for *L*/*D* = 100 and *L*/*D* = 20, which are shown in Fig. 3 together with that of *L*/*D* → ∞. As far as we are aware, SPT has not yet been applied to predict the phase diagram of hard rods in a quadrupole field. We conclude from this figure that the phase diagram expressed in terms of the scaled concentration *c* and the dimensionless field strength *K* only starts to deviate significantly when the aspect ratio is smaller than roughly one hundred. However, the external field dependence seems rather insensitive to a variation of aspect ratio of the particles. Indeed, the critical and tricritical field strengths vary only by a few percent if the aspect ratio decreases from infinity down to twenty. At *K* = 0, our results show excellent agreement with earlier simulations and numerical work.^72^

To apply SPT to our calculation of the cluster size, we closely follow the procedure presented in Finner *et al.*^66^ We rescale our contact volume with the same parameter *Γ* that we used to rescale our excluded volume, given by eqn (10), and explicitly include the contributions due to the end-effects to the excluded volume. The closure of the connectedness Ornstein–Zernike eqn (5) now becomes11
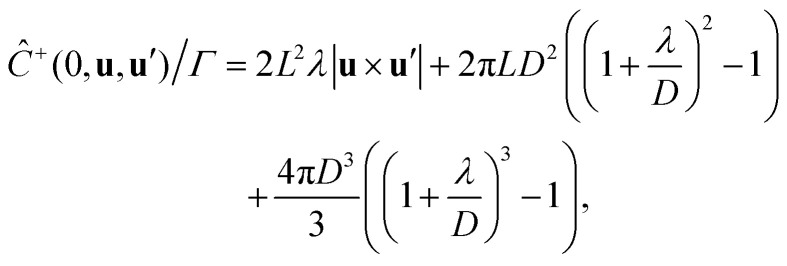
for **q***L* → **0**, where the second and third terms account for the effects of the hemispherical end-caps.

That we should rescale the closure (11) with the factor *Γ* does not follow from rigorous theoretical arguments. However, it may be justified on the grounds that it makes sense to make use of the same renormalisation of the excluded volume term of the free energy as that in the direct connectedness function, which in effect is a connectedness volume.^66^ Perhaps more convincingly, it turns out that this approximation yields very accurate results for aspect ratios *L*/*D* > 10.^66^

**Fig. 4:**
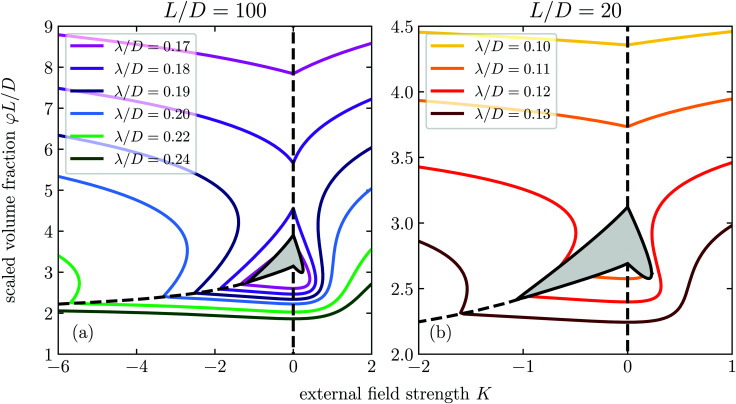
Percolation diagram for spherocylinders with aspect ratio *L*/*D* = 100 (a) and *L*/*D* = 20 (b) as a function of the external field strength *K* and concentration *c* for different values of the connectedness criterion *λ*/*D*. The hatched region denotes the region of phase coexistence.

By the same methods described in Section 3, we solve the connectedness Ornstein–Zernike equation and find the cluster size as function of the volume fraction *φ*, aspect ratio *L*/*D*, connectedness criterion *λ*/*D*, and external field strength *K*. We summarise our findings in the percolation diagrams shown in Fig. 4, in which we plot for the aspect ratios *L*/*D* = 100 and *L*/*D* = 20 the percolation threshold for a range of different connectedness criteria as a function of the external field strength. The percolation thresholds are expressed in terms of the product *φL*/*D* of the volume fraction and the aspect ratio, which in the slender particle limit becomes the definition of our parameter *c*. We recall that the diagram for *L*/*D* → ∞ is shown in Fig. 2.

Comparing Fig. 2 with Fig. 4, we notice that the finite particle slenderness introduces an additional percolation transition at high concentrations. This agrees with the findings of Finner *et al.*^45^ for the case *K* ≥ 0. This is not hugely surprising, since, after all, a finite dispersion with sufficiently many particles must percolate even if perfectly aligned, although it may be superseded by a transition to a smectic or crystalline phase.

Intuitively, the location of this high concentration percolation threshold must strongly depend on the aspect ratio of the particles, and this is indeed what we find. Apart from this additional percolation threshold, our results show that the topology of the percolation diagram does not change as the aspect ratio decreases down to *L*/*D* = 20. However, if we decrease the degree of particle anisometry further, so below *L*/*D* = 20, we find that the percolation islands and peninsulas disappear entirely (results not shown). Notice that for aspect ratios near *L*/*D* = 5 we expect the nematic transition to disappear altogether and be replaced by a transition to a smectic or crystalline state.^73^ For longer particles, the nematic–smectic A transition occurs at volume fractions in the range *φ* ∈ (0.4, 0.6).^74–77^ In particular, this means that this transition occurs well above the densities considered in Fig. 4 and therefore should leave the reported phenomenology intact.

For spherical particles, so for *L*/*D* = 0, the diagram should lose its dependence on the external field completely, as the connectedness and excluded volumes become independent of the particle orientations. In this case, the concentration at which percolating clusters appear depends only on the ratio between the connectivity criterion and the particle diameter. See, *e.g.*, Miller.^78^

Now that we have discussed under what conditions materials percolate, it turns out to be instructive to investigate the shape of the clusters near and away from the percolation threshold. For this, we need to a have closer look at the theory for finite values of the wave vector **q**. As we shall see, subcritical clusters in the biaxial nematic state can be highly triaxial.

## 5 Cluster shape

In order to analyse the cluster shape, we rely on the methods employed in earlier work.^44,45^ We do so in the Onsager limit, valid for particles with very high aspect ratios and expect that finite aspect ratio corrections to this procedure do not qualitatively change our main findings. We quantify the shape of the clusters by evaluating the **q***L* → 0 behaviour of the connectedness structure factor *S*^+^(**q**). From the structure factor, we are then able to identify the correlation lengths.

As a first step, we write down a formal expansion of the structure factor for small **q***L*12
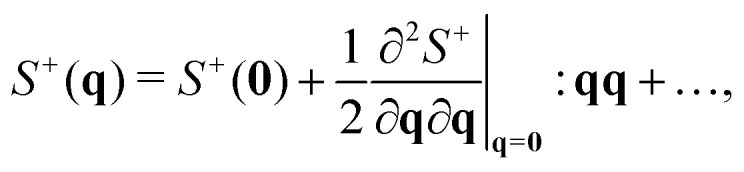
where, as usual, the double dot product is defined as 
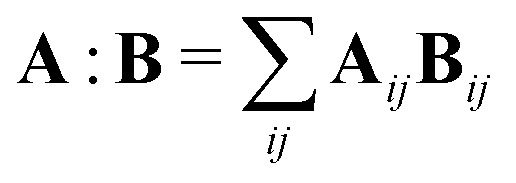
, in which **A** and **B** represent matrices. The diadic products are defined as (**ab**)_*ij*_ = **a**_*i*_**b**_*j*_, where **a** and **b** are vectors. Since the rods are inversion symmetric, our theory must be invariant under the transformation **q** → −**q**. This implies that the odd orders and cross terms in the expansion must vanish and therefore only the second order diagonal terms remain in eqn (12). From their dimensions these coefficients can be interpreted as correlation lengths, and therefore they must provide us with information about the shape of clusters.

In accordance with earlier work of Finner *et al.*,^45^ we define the correlation lengths as13
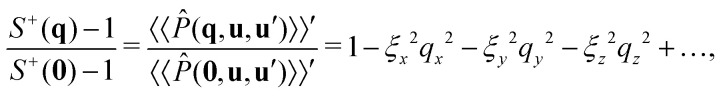
where *ξ*_*x*_ is the correlation length in the *x*-direction, and so on. In the isotropic phase, all correlation lengths are equal, whilst in the uniaxial phases two out of the three are equal. We interpret these correlation lengths as measures of the average dimensions of a cluster.

To calculate the correlation lengths, we follow Finner *et al.*^45^ and find an expression for the leading order anisotropic term in the expansion of the pair connectedness function14
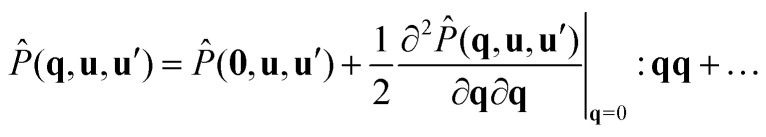
The tensor 
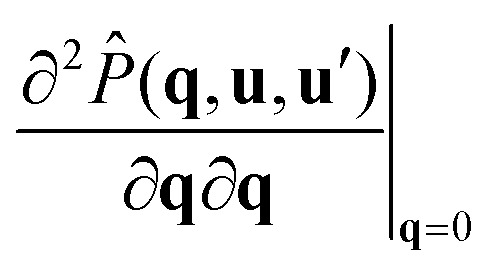
 contains all the information required to find the correlation lengths. By substituting eqn (14) into eqn (13), we find‡‡This expression corrects a typographical error in earlier work.^45^ The typographical error does not affect any of the results presented in that work.15
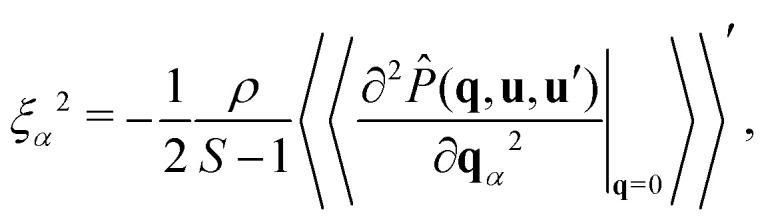
where the Greek index *α* can denote any of the Cartesian coordinates.

As eqn (15) suggests, we can restrict our calculation to finding the tensor 
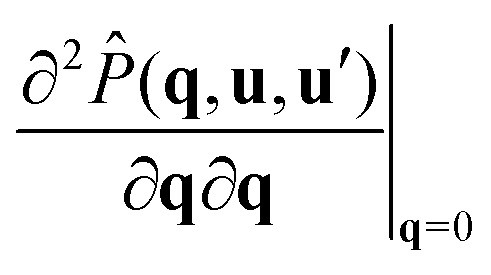
 instead of the full *P̂*(**q**, **u**, **u**′). For this, we take the second derivative of the connectedness Ornstein–Zernike eqn (5) and evaluate it in the limit **q** → **0**. Because the first derivatives of both *P̂* and *Ĉ*^+^ vanish at **q** = **0**, this simplifies to16
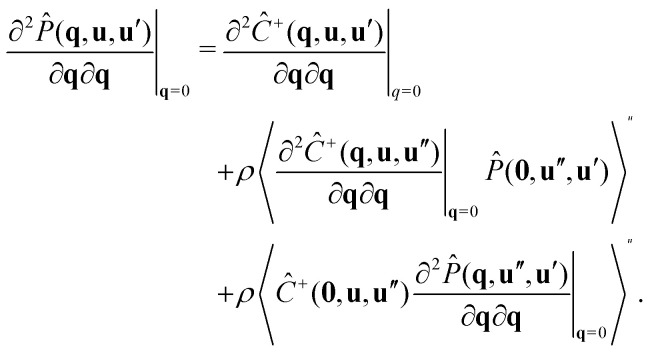
Using the fact that^‡^
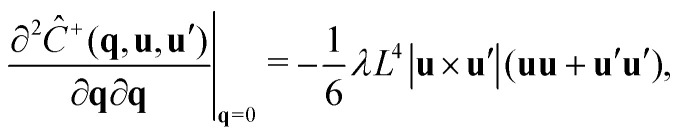
 we obtain a closed equation for the tensor 
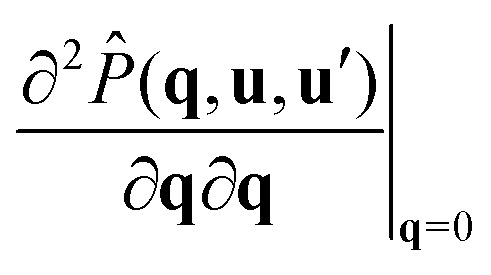
, which reads17
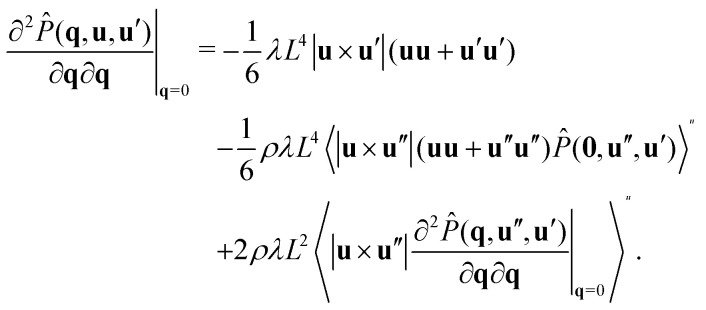
Similarly to our treatment of the **q***L* = **0** version of the connectedness Ornstein–Zernike equation, we can simplify the computation by pre-averaging this equation over **u**′, and solving the result one matrix element at a time, as the elements are not coupled. The correlation lengths are now easily evaluated with eqn (15). We numerically find that the off-diagonal elements do vanish, as demanded by our symmetry arguments.

**Fig. 5:**
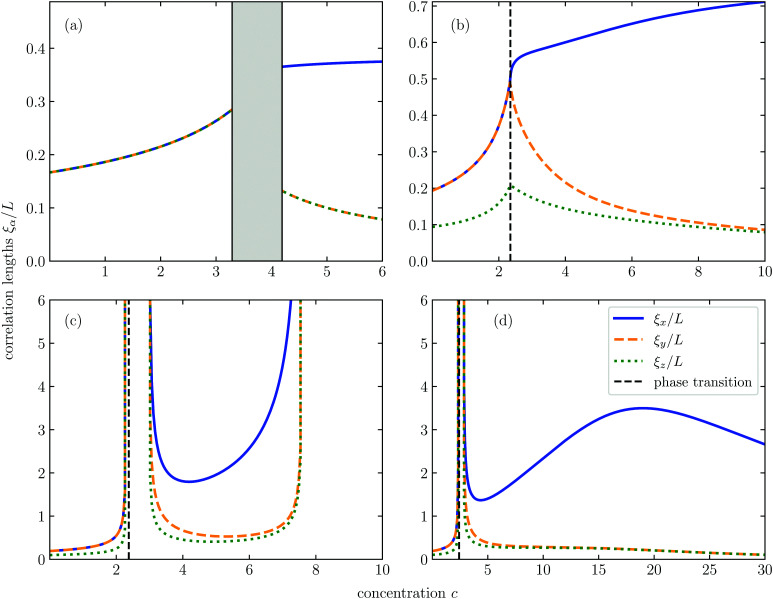
Correlation lengths as a function of the concentration along vertical slices in Fig. 2. The first and second order phase transitions are indicated in black. The figures correspond to the slices across the isotropic–nematic transition at *K* = 0, *λ*/*D* = 0.1 (a), and across the antinematic–biaxial nematic transition at *K* = −5, *λ*/*D* = 0.2 (b); *K* = −4.5, *λ*/*D* = 0.24 (c), and *K* = −4, *λ*/*D* = 0.23 (d). We see a wide variety of behaviour across this transition, depending on the field strength and connectivity length.

In Fig. 5, we plot the various correlation lengths *ξ*_α_/*L* for different connectivity lengths *λ*/*D* and field strengths *K*, as a function of the dimensionless concentration *c*. As we have seen in Section 3, for sufficiently small values of *λ*/*D*, no percolation can occur. Indeed, the correlation lengths in this regime remain finite, as shown in Fig. 5a for *K* = 0 and *λ* = 0.1*D*. As expected, we only find one independent correlation length in the isotropic phase, and two in the nematic phase of which the one along the director is larger than that perpendicular to it. This means that in the nematic phase the particle clusters become elongated, that is, longer than they are wide.

For *K* > 0, clusters are always elongated, the degree to which depends on the strength of the field and the concentration. For a discussion of the cluster shapes for *K* > 0 we refer to the work of Finner *et al.*,^45^ and in the remainder of this section we focus attention exclusively on the case *K* < 0.

If, for instance, we choose *K* = −5 and *λ* = 0.2*D*, then again there is no percolation for any concentration as Fig. 5b shows. However, in the dilute phase, the correlation length parallel to the field axis is smaller than the correlation lengths perpendicular to it, indicating that the clusters are oblate rather than prolate.

As we cross the continuous phase transition line indicated by black dashed lines in Fig. 5, the induced biaxiality of the orientational distribution function makes itself felt in a cluster that is longer along the director (which is defined parallel to the *x*-axis) than along the field direction (parallel to the *z*-axis). In the direction perpendicular to both the external field and the nematic director (along the *y*-axis), the clusters are wider than along the field direction.

Notice that the degree of triaxiality of the clusters decreases with increasing concentration in the biaxial nematic phase, because both correlation lengths perpendicular to the director field become increasingly equal. This is caused by the fact that, relatively speaking, the impact of the (triaxiality-driving) external field decreases compared to that of the hard-core interactions that favour uniaxial order.

To illustrate what happens to the cluster shape if we encounter a sequence of re-entrance phenomena, we show the correlation lengths for *K* = −4.5 and *λ* = 0.24*D* in Fig. 5c and for *K* = −4 and *λ* = 0.233*D* in Fig. 5d as a function of the concentration *c*. In Fig. 5c we take a vertical cut through the state diagram of Fig. 2 on the left of the tricritical point. This means that we again cross the continuous phase transition between the antinematic and biaxial nematic. Before we cross this transition, however, a percolating cluster emerges, characterised by diverging correlation lengths in all three directions. This percolating state does not disappear until well into the biaxial nematic phase. Interestingly, even more deeply in the biaxial nematic phase, percolation is restored.

In Fig. 5d, we illustrate a superficially counter-intuitive phenomenon, namely, that further increasing the concentration of particles deep in the biaxial nematic phase does not always lead to larger clusters. It is important to realise that the cluster dimensions are directly linked to the distance from the percolation threshold in *K*, *c* parameter space. What Fig. 5d shows, is that for the chosen parameters, we approach a percolation threshold with increasing concentration, but at higher concentrations move away from it again. The decrease of the cluster size at very high concentrations is in effect caused by the increased alignment of the particles.

On the surface of it, this mirrors what happens in the absence of a field.^65^ However, we conclude from Fig. 5d, that only one of the correlation lengths increases when approaching the percolation threshold, while the others do not. This is qualitatively different from the zero-field case, where the ratio of the correlation lengths grows linearly with the concentration.^65^ We have no explanation for why in this particular case the behaviour of *ξ*_*x*_ seems to be decoupled from the that of *ξ*_*y*_ and *ξ*_*z*_.

This leads us to the conclusions of this paper, in which we summarise and briefly discuss our findings.

## 6 Conclusions

In summary, we have numerically investigated the effect of external-field-induced antinematic and paranematic order on the percolation threshold and cluster shape in dispersions of spherocylindrical particles, extending the earlier work of Finner *et al.*^45^ We have applied Scaled Particle Theory corrections to approximately incorporate the impact of higher order virial terms in the Onsager free energy, and used a similar approximation for the direct connectedness function. This has allowed us to generalise our findings to rods with aspect ratios of down to approximately *L*/*D* = 20.

For connectivity lengths satisfying *λ* > 0.15*D*, we find that percolation islands form in the percolation diagram, meaning that upon an increase of the concentration, percolating clusters can form and break down subsequently. For particles with finite aspect ratios, a high density percolation threshold always exists, as one would expect. In the Onsager limit, in which end-effects are neglected, this high density percolation threshold does not exist.

We find that if we increase the connectivity length, the percolation islands grow in size, and eventually fan out, connecting to the high density threshold. If the connectivity length is sufficiently large, percolation cannot be lost beyond the percolation threshold, *i.e.*, and the re-entrance phenomenon disappears.

As to sub-critical cluster shapes, we confirm earlier findings that clusters are spherical in the isotropic state but elongated in the uniaxially aligned phases. Moreover, across the continuous antinematic–biaxial nematic phase transition occurring subject to disorienting fields, the clusters shift in shape from oblate spheroidal to triaxial prolate ellipsoidal according to our findings. We speculate that the highly anisometric shape of clusters in these phases might be exploitable to manufacture materials that have anisotropic conductivity properties.^79^

We expect our predictions to become less accurate for small particle aspect ratios. Hence, it would be useful to verify our calculations by means of simulations. To the best of our knowledge, such simulations have not yet been conducted—at least not under conditions of full thermodynamic equilibrium.

Interestingly, our results in the disorientational regime do show a surprising qualitative resemblance with the percolation diagrams obtained by dynamical simulations of nanorods subject to shear flow.^80^ Indeed, if we compare Fig. 5b and 6b of Kwon *et al.*^80^ with our Fig. 2, we conclude that a shear field has a similar impact on the topology of the percolation diagram as a disorientational field. In particular, in both multiple re-entrance phenomena occur depending on the field strength and the aspect ratio of the particles. Since Kwon and coworkers explain this re-entrant percolation behaviour in terms of localised aggregation of particles rather than of particle alignment, the correspondence might be coincidental.

Ideally, we would also like to compare our predictions with experimental work. We have not been able to find any such work in the literature, but are aware of the experimental work of Yuan *et al.*^36^ highlighting the competition between percolation and the nematic transition in dispersions of graphene flakes, which does lend some support to some of our findings.

Similar experiments have not yet been performed involving, *e.g.*, carbon nanotubes, and for these it is difficult to envisage an external field that is of the planar type, other than an extensional flow field imposed in a four-roll-mill setup.^47^ We realise that such experiments are not trivial to conduct, as it is a major challenge to disperse carbon nanotubes homogeneously in any type of fluid, except perhaps chlorosulfonic acid.^81^

From an application point of view, the situation is even more complex due to the impact of the manufacturing process, including spin coating and shear mixing, on the orientational order in the particle dispersions.^29,82^ Even in controlled laboratory experiments, external electric, magnetic or flow fields are difficult to avoid, and our results show that the effects of such weak external fields on the percolation threshold can be very significant. We believe that this can in part explain the huge scatter in the experimental measurements of the conductivity of nanocomposite materials that seem superficially very similar.^26^

## Author contributions

Ilian Pihlajamaa: formal analysis, investigation, methodology, software, validation, visualisation, writing – original draft. René de Bruijn: conceptualisation, formal analysis, supervision, validation, writing – review and editing. Paul van der Schoot: conceptualisation, formal analysis, funding acquisition, methodology, project administration, resources, supervision, writing – review and editing.

## Conflicts of interest

There are no conflicts to declare.

## Supplementary Material
